# Dr. Crawford W. Long

**Published:** 1902-09

**Authors:** 


### DR. CRAWFORD W. LONG.

IF the primacy of greatness be determined by the measure of good bestowed on the human race, certainly there are few greater names in the world's history than that of Crawford Long. The appended notice from the chairman of the Long Memorial

Committee will explain the object of the commission. This is a notable cause, and one to which, prompted by motives of professional pride, patriotism and gratitude, the contributions should be given unhesitatingly and freely. It is the confident belief of those concerned that the amount will soon be raised, as it is small indeed contrasted with the debt that so many owe to him whose effigy is to be preserved at the National Capitol.

THE CRAWFORD W. LONG MEMORIAL FUND.

In the Capitol at Washington there is a hall generally known as the Hall of Fame, a place of honor in which there are to be placed statues of two of the distinguished men of each State.

The last Georgia Legislature appointed a commission to select the names of the two citizens of this State whose deeds made them worthy of this distinction.

From the first the name of Dr. Crawford W. Long, the discoverer of anesthesia, that beneficent agent that has acquired such power to save life and alleviate human suffering, was the one most prominently discussed as being worthy of the honor.

The last meeting of the Medical Association of Georgia appointed a committee of one member from each Congressional District, known as the "Crawford W. Long Memorial Committee." The object of the committee was to advocate the selection of Dr. Long, and succeeding in this to raise the funds necessary to erect his statue.

The first is an actuality. The legislative committee unanimously selected Dr. Long, and appointed a committee of three—Judge George Hillyer of Atlanta, Judge A. L. Miller of Macon, and Mr. A. L. Hull of Athens—to confer with the Long Memorial Committee in regard to ways and means.

A suitable statue will cost about ten thousand dollars, and it has been decided to raise this amount by popular subscription ; not only from the medical profession, who are actually interested, but also from the laity. For there are few families in which there is not some one near and dear who has felt the wonderful relief of this agent, and who will be willing to contribute their mite in aiding to honor the genius of the discoverer of anesthesia.

The committee asks the aid of every physician.

They would like for each physician not only to subscribe to the fund, but to make himself a committee of one to get as many subscriptions as he can from his friends and patrons. If each physician will interest himself in this way the funds can easily be raised.

The subscriptions can be sent to any member of the committee, which is as follows:

Dr. W. W. Owens, Savannah, Ga., First District.
Dr. J. D. Chason, Bainbridge, Ga., Second District.
Dr. Alexander Mack, Hawkinsville, Ga., Third District.
Dr. F. M. Ridley, LaGrange, Ga., Fourth District.
Dr. F. W. McRae, Atlanta, Ga., Fifth District.
Dr. Howard J. Williams, Macon, Ga., Sixth District.
Dr. R. M. Harbin, Rome, Ga., Seventh District.
Dr. Samuel Benedict, Athens, Ga., Eighth District.
Dr. L. G. Hardman, Harmony Grove, Ga., Ninth District.
Dr. J. B. Morgan, Augusta, Ga., Tenth District.
Dr. Chas. Hicks, Dublin, Ga., Eleventh District.
Dr. W. F. Westmoreland, Chairman, Atlanta, Ga.

---

THE proprietor of a nostrum of wide exploitation—Swamp Root—displays the most cheerful effrontery that has yet been manifested in the wild domain of quackery. Not content with offending the vision of every medical reader of the daily papers with the pseudo-science and fluffy nonsense of his pretensions and testimonials, he has had the eburnated nerve to send samples of his "discovery" to physicians themselves, with a precursory letter which offers light to those who yet grope in the darkness of renal therapeutics. This is a display of derisive daring under the guns that in any other setting would meet with applause. We cannot but admire the cool courage of this Cagliostro. It is usual for quacks to acknowledge to physicians the thinness of their pretensions and attempt to extenuate them by reference to the desire of the public to be deceived, but this swamp angel carries his goods for display beneath the horns of the altar. There may perhaps be some of the brethren so weak in the faith that they will add their own to the clerical and aged spinster collection of testimonials, but the sample sent us was returned to the closest substitute at hand for the native habitat of the "discovery"—the spittoon.

It seems that in this day of false cults and organized charlatanry the old-fashioned quack, with his purely vegetable cure-all, is being forced to seek protection by his traditional enemy, the doctor. Christian Science, Osteopathy and kindred evils have run him into a *cul-de-sac*, from which he seeks to escape by craving for aid from the medical profession, as being more nearly affiliated with him than are the apostles of his own creed. "And these in turn have smaller fleas to bite 'em, and so on, *ad infinitum*."

---

KATHERINE L. BALL, of San Francisco, is dead of starvation, resulting from self-imposed fasting. Mrs. Ball was about fifty years old and was exceedingly stout. Her weight shortly before her fast commenced was 250 pounds, and her increasing bulk caused her much worry. On the advice of Dr. J. C. Anthony she undertook a fast that was to last 50 days. At the end of 15 days Mrs. Ball had lost 40 pounds, but she felt strong and had not the slightest craving for food. On that day, too, she climbed three flights of stairs without stopping for breath, a feat that she had not accomplished in many years. Until the twenty-first day Mrs. Ball's improvement continued. She became stronger and more active. She talked constantly of her marvelous fast and often expressed her intention of keeping it up for 50 days. But the endurance of her constitution had been put under too severe a strain. On the forty-fifth day of the fast members of the family sent for another physician. She was induced to break her fast by taking light food and strengthening medicines. Trained nurses were employed and strenuous efforts were made to restore her strength, but failed. The body is that of a frail, emaciated woman. At the time of her death Mrs. Ball weighed not more than 120 pounds.—*St. Louis Med. Review*.

